# Patient-Active Control of a Powered Exoskeleton Targeting Upper Limb Rehabilitation Training

**DOI:** 10.3389/fneur.2018.00817

**Published:** 2018-10-11

**Authors:** Qingcong Wu, Xingsong Wang, Bai Chen, Hongtao Wu

**Affiliations:** ^1^College of Mechanical and Electrical Engineering, Nanjing University of Aeronautics and Astronautics, Nanjing, China; ^2^College of Mechanical Engineering, Southeast University, Nanjing, China

**Keywords:** upper limb exoskeleton, robot-assisted, rehabilitation training, patient-active control, intention-driven, virtual environment

## Abstract

Robot-assisted therapy affords effective advantages to the rehabilitation training of patients with motion impairment problems. To meet the challenge of integrating the active participation of a patient in robotic training, this study presents an admittance-based patient-active control scheme for real-time intention-driven control of a powered upper limb exoskeleton. A comprehensive overview is proposed to introduce the major mechanical structure and the real-time control system of the developed therapeutic robot, which provides seven actuated degrees of freedom and achieves the natural ranges of human arm movement. Moreover, the dynamic characteristics of the human-exoskeleton system are studied via a Lagrangian method. The patient-active control strategy consisting of an admittance module and a virtual environment module is developed to regulate the robot configurations and interaction forces during rehabilitation training. An audiovisual game-like interface is integrated into the therapeutic system to encourage the voluntary efforts of the patient and recover the neural plasticity of the brain. Further experimental investigation, involving a position tracking experiment, a free arm training experiment, and a virtual airplane-game operation experiment, is conducted with three healthy subjects and eight hemiplegic patients with different motor abilities. Experimental results validate the feasibility of the proposed scheme in providing patient-active rehabilitation training.

## Introduction

Stroke is a severe neurological disease caused by the blockages or rupture of cerebral blood vessels, leading to significant physical disability and cognitive impairment (1, 2). The recent statistics from the World Health Organization indicate that worldwide 15 million people annually suffer from the effect of stroke, and more than 5 million stroke patients survive and, however, require a prolonged physical therapy to recover motor function. Recent trends predict increased stroke incidence at younger ages in the upcoming years (3, 4). Approximately four-fifths of all survived stroke patients suffer from the problems of hemiparesis or hemiplegia and, as a result, have difficulties in performing activities of daily living (ADL). Stroke causes tremendous mental and economic pressure on the patients and their families (5). Medical research has proved that, owing to the neural plasticity of the human brain, appropriate rehabilitation trainings are beneficial for stroke survivors to recover musculoskeletal motor abilities. Repetitive and task-oriented functional activities have substantial positive effects on improving motor coordination and avoiding muscle atrophy (6, 7). Traditional stroke rehabilitation therapy involves many medical disciplines, such as orthopedics, physical medicine, and neurophysiology (8, 9). Physiotherapists and medical personnel are required to provide for months one-on-one interactions to patients that are labor intensive, time consuming, patient-passive, and costly. Besides, the effectiveness of traditional therapeutic trainings is limited by the personal experiences and skills of therapists (10, 11).

In recent decades, robot-assisted rehabilitation therapies have attracted increasing attention because of their unique advantages and promising applications (12, 13). Compared with the traditional manual repetitive therapy, the use of robotic technologies helps improve the performance and efficiency of therapeutic training (14). Robot-assisted therapy can deliver high-intensive, long-endurance, and goal-directed rehabilitation treatments and reduce expense. Besides, the physical parameters and the training performance of patients can be monitored and evaluated via built-in sensing systems that facilitate the improvement of the rehabilitation strategy (15, 16). Many therapeutic robots have been developed to improve the motor functions of the upper extremity of disabled stroke patients exhibiting permanent sensorimotor arm impairments (17). The existing robots used for upper limb training can be basically classified into two types: end-point robots and exoskeleton robots. End-point robots work by applying external forces to the distal end of impaired limbs, and some examples are MIME (18), HipBot (19), GENTLE/s (20), and TA-WREX (21). Comparatively, exoskeleton robots have complex structures similar to anatomy of the human skeleton; some examples of such robots are NMES (22), HES (23), NEUROExos (24), CAREX-7 (25), IntelliArm (26), BONES (27), and RUPERT (28). The joints of the exoskeleton need to be aligned with the human anatomical joints for effective transfer of interactive forces.

The control strategies applied in therapeutic robots are important to ensure the effectiveness of rehabilitation training. So far, according to the training requirement of patients with different impairment severities, many control schemes have been developed to perform therapy and accelerate recovery. Early rehabilitation robot systems implemented patient-passive control algorithms to imitate the manual therapeutic actions of therapists. These training schemes are suitable for patients with severe paralysis to passively execute repetitive reaching tasks along predefined trajectories. Primary clinical results indicate that patient-passive training contributes to motivating muscle contraction and preventing deterioration of arm functions. The control of the human–robot interaction system is a great challenge due to its highly nonlinear characteristics. Many control algorithms have been proposed to enhance the tracking accuracy of passive training, such as the robust adaptive neural controller (29), fuzzy adaptive backstepping controller (30), neural proportional–integral–derivative (PID) controller (31), fuzzy sliding mode controller (32), and neuron PI controller (33).

The major disadvantage of patient-passive training is that the active participation of patients is neglected during therapeutic treatment (34). Several studies suggest that, for the patients who have regained parts of motor functions, the rehabilitation treatment integrated with the voluntary efforts of patients facilitates the recovery of lost motor ability (35). The patient-active control, normally referred as patient-cooperative control and assist-as-needed control, is capable of regulating the human–robot interaction depending on the motion intention and the disability level of patients. Keller et al. proposed an exoskeleton for pediatric arm rehabilitation. A multimodal patient-cooperative control strategy was developed to assist upper limb movements with an audiovisual game-like interface (36). Duschauwicke et al. proposed an impedance-based control approach for patient-cooperative robot-aided gait rehabilitation. The affected limb was constrained with a virtual tunnel around the desired spatial path (37). Ye et al. proposed an adaptive electromyography (EMG) signals-based control strategy for an exoskeleton to provide efficient motion guidance and training assistance (38). Oldewurtel et al. developed a hybrid admittance–impedance controller to maximize the contribution of patients during rehabilitation training (39). Banala et al. developed a force-field assist-as-need controller for intensive gait rehabilitation training (40). However, there are two limitations in the existing patient-cooperative control strategies. Firstly, the rehabilitation training process is not completely patient-active, as the patient needs to perform training tasks along a certain predefined trajectory. Secondly, existing control strategies are executed in self-designed virtual scenarios that are generally too simple, rough, and uninteresting. Besides, applying a certain control strategy to different virtual reality scenarios is difficult.

Taking the above issues into consideration, the main contribution of this paper is to develop a control strategy for an upper limb exoskeleton to assist disabled patients in performing active rehabilitation training in a virtual scenario based on their own active motion intentions. Firstly, the overall structure design and the real-time control system of the exoskeleton system are briefly introduced. A dynamic model of the human–robot interaction system is then established using the Lagrangian approach. After that, an admittance-based patient-active controller combined with an audiovisual therapy interface is proposed to induce the active participation of patients during training. Existing commercial virtual games without a specific predetermined training trajectory can be integrated into the controller via a virtual keyboard unit. Finally, three types of experiments, namely the position tracking experiment without interaction force, the free arm movement experiment, and the virtual airplane-game operation experiment, are conducted with healthy and disabled subjects. The experimental results demonstrate the feasibility of the proposed exoskeleton and control strategy.

## Materials and methods

### Exoskeleton robot design

**Figure 1:**
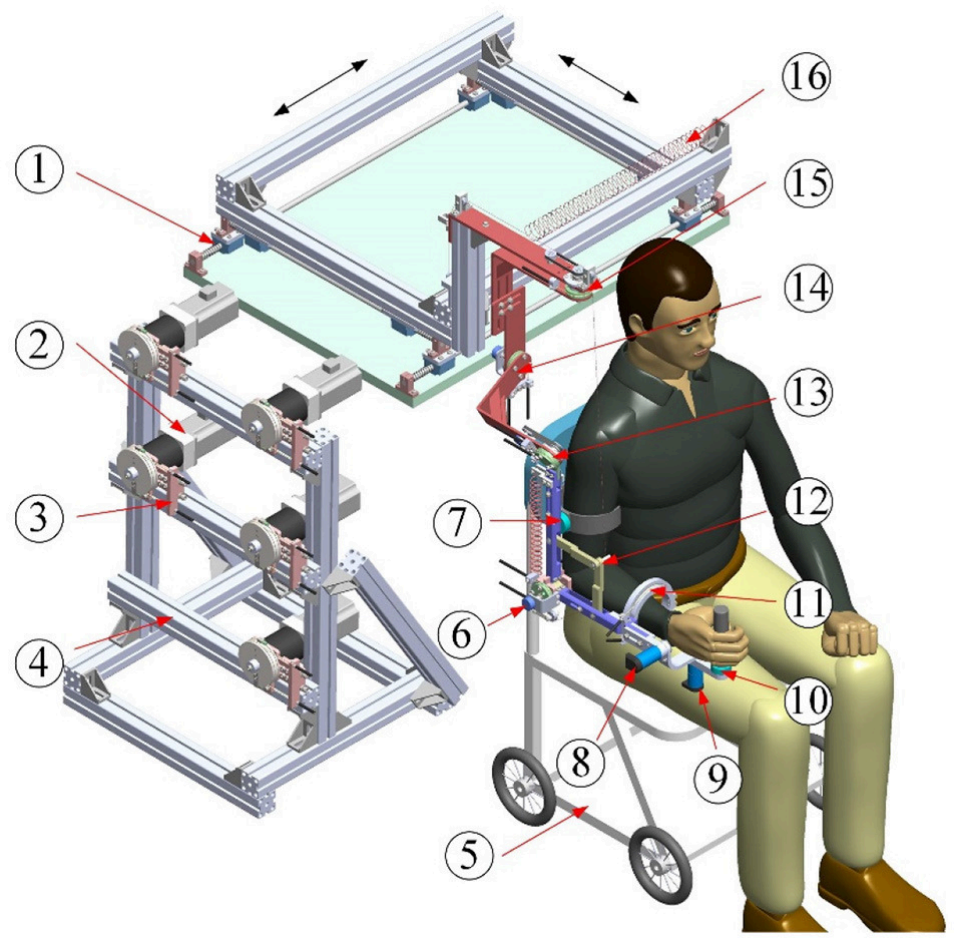
Architecture of upper limb rehabilitation exoskeleton (1-Self-aligning platform; 2-AC servo motor; 3-Bowden cable components; 4-Support frame; 5-Wheelchair; 6-Elbow flexion/extension; 7-Proximal force/torque sensor; 8-Wrist flexion/extension; 9-Wrist ulnal/radial deviation; 10-Distal force/torque sensor; 11-Forearm pronation/supination; 12-Auxiliary links; 13-Shoulder flexion/extension; 14-Shoulder abduction/adduction; 15-Shoulder internal/external; 16-Free-length spring).

The architecture of the proposed exoskeleton is shown in Figure 1. This wearable force-feedback exoskeleton robot has seven actuated degrees of freedom (DOFs) and two passive DOFs covering the natural range of movement (ROM) of humans in ADL. The robot has been designed with an open-chain structure to mimic the anatomy of the human right arm and provide controllable assistance torque to each robot joint. There are three actuated DOFs at the shoulder for internal/external rotation, abduction/adduction, and flexion/extension; two DOFs at the elbow for flexion/extension and pronation/supination; and two DOFs at the wrist for flexion/extension and ulnal/radial deviation. Besides, since the center of rotation of the glenohumeral joint varies with the shoulder girdle movement, the robot is mounted on a self-aligning platform with two passive translational DOFs to compensate the human–robot misalignment and to guarantee interaction comfort.

**Figure 2:**
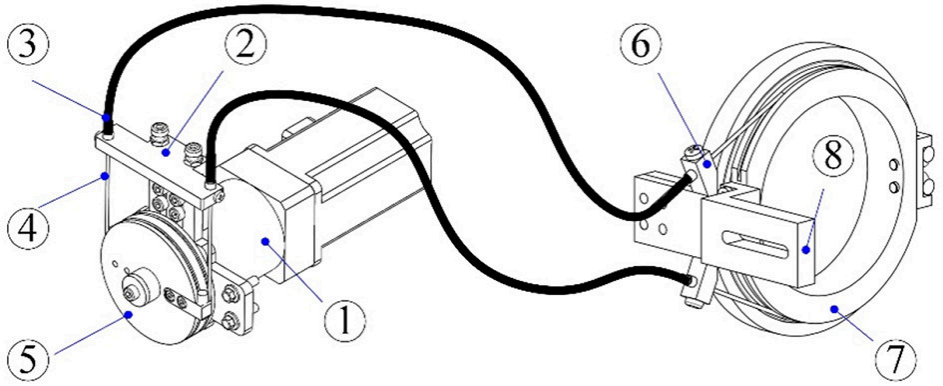
Schematic of the Bowden-cable actuation system (1-Servo motor and planetary gear reducer; 2-Pretension device; 3-Outer sheath; 4-Inner cable; 5- Proximal pulley; 6-Cable supports; 7-Distal pulley; 8-Exoskeleton link).

The lengths of the upper arm and the forearm as well as the shoulder height of the exoskeleton can be adjusted to satisfy the dimensions of patients with body heights in the range of 1.6 m to 1.9 m. The revolute robot joints at the shoulder and the elbow are actuated by the flexible Bowden-cable actuation systems located on the remote support frame (41). The schematic of the Bowden-cable actuator is depicted in Figure 2. It mainly consists of a servo AC motor (SGMAV-04A, YASKAWA) combined with a planetary gear reducer (Yantong, transmission ratio: 40) used to produce a maximum torque of 35 Nm, two bendable Bowden-cable transmission units used to transmit driving torque from the proximal pulley to the distal pulley, and a pretension device used to adjust cable pretension and eliminate the slacking problem resulting from cable elasticity. In addition, the wrist joints of the exoskeleton are actuated by two high-precision coreless DC servo motors (JG-37, ASLONG).

A passive gravity-compensation mechanism made up of two zero free-length springs and two auxiliary parallel links is integrated into the exoskeleton to balance the gravity of the entire system [detailed description can be found in (42)]. The configuration of the exoskeleton is measured by using a rotary potentiometer (WDJ22A-10K) encapsulated in each robot joint. Two six-axis force/torque (F/T) sensors (NANO-25, ATI) are located at the upper arm and the end-effector to acquire the interaction forces between the robot and the operator. The custom Velcro fasteners attached to the upper arm and the forearm of the exoskeleton facilitate the connection and separation between the human arm and the robot. For safety, the available ROM of each actuated joint is limited via a mechanical stop to avoid collisions and excessive motion. Two dead-man buttons are available to both the patient and physiotherapists to immediately shut down the motor power in the case of an emergency.

### Electrical control system

**Figure 3:**
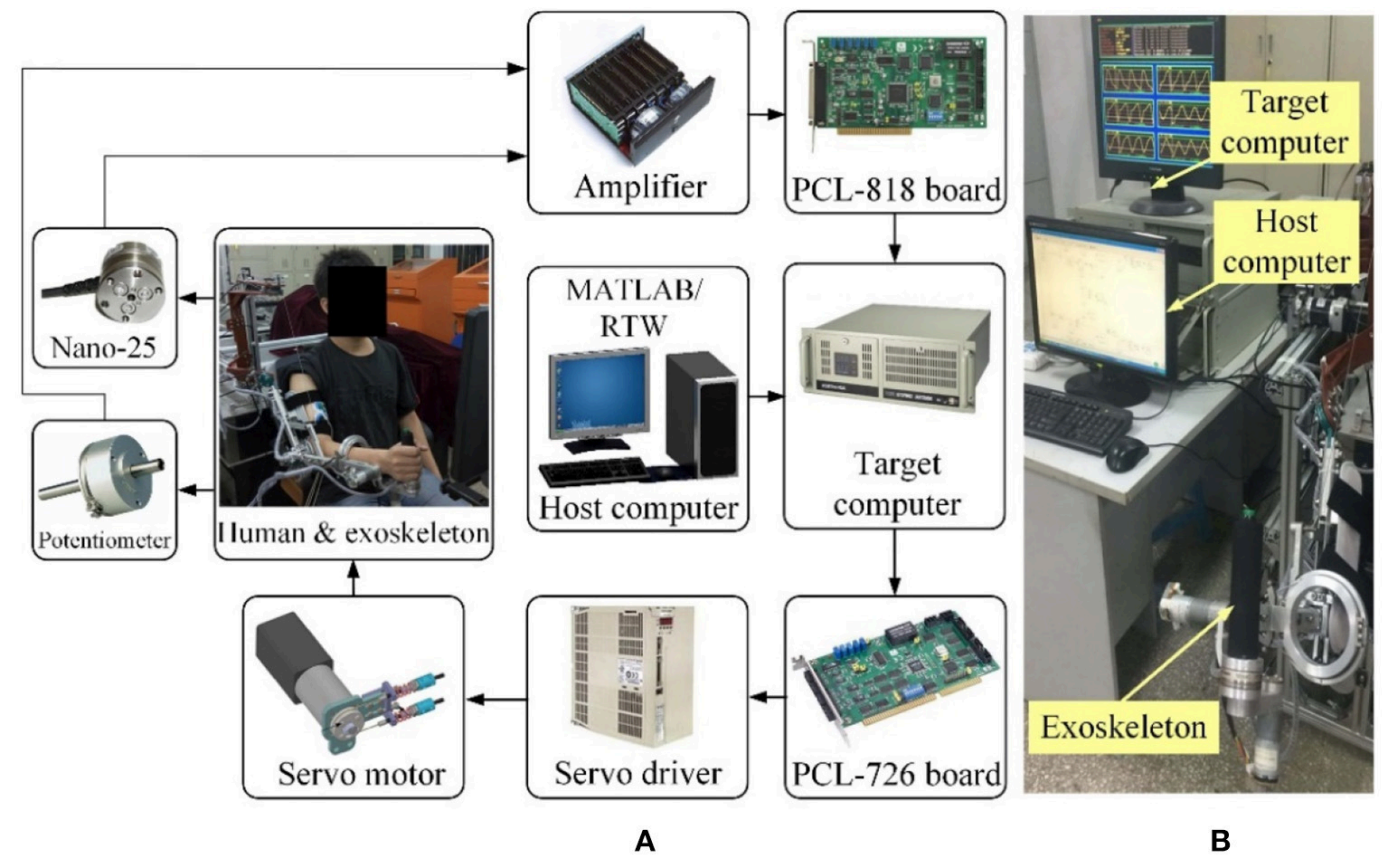
The MATLAB/Real-Time-Workshop/xPC control system. **(A)** Hardware architecture diagram. **(B)** Experimental setup.

The closed-loop real-time control system of the therapeutic exoskeleton is built in the MATLAB/Real-Time-Workshop (RTW) environment with a hierarchical architecture, as shown in Figure 3. Two industrial personal computers (IPC-610H, Advantech Inc.) are used as the host and target computers. The host computer is capable of transforming the Simulink control model to the executable codes, while the target computer takes charge of implementing the embedded targeted codes and regulating the operation of the robot system. The analog feedback signals from the potentiometers and F/T sensors are amplified by power amplifiers (LKM-66, FMS) and acquired via three industrial analog-to-digital converters (PCL-818, Advantech Inc.). The control algorithm running in the target computer is converted into analog output signals by two industrial digital-to-analog cards (PCL-726, Advantech Inc.) The output analog signals are transmitted to the servo drivers to control servo motors with a sampling rate of 1 kHz.

### Dynamics modeling

It is important to analyze the dynamic characteristics of the exoskeleton equipped on the right arm of the operator to improve the control system performance. The overall dynamic model of the exoskeleton with human–robot interaction forces can be established based on the Lagrangian approach as follows:

(1) $$\text{M}(\theta )\overset{..}{\theta }+\text{V}(\theta ,\dot{\theta })+\tau_{\text{f}}(\theta ,\dot{\theta })+{\text{D}}_{\text{u}}\text{−}{\text{J}}_{1}^{\text{T}}(\theta )\Gamma_{1}-{\text{J}}_{2}^{\text{T}}(\theta )\Gamma_{2}=\tau ,$$

where $\theta ,\overset{\cdot }{\theta },\text{and }\ddot{\theta }$ denote the vectors of generalized positions, velocities, and accelerations of actuated joints. **M**(θ) represents the symmetric positive-defined system inertia matrix. $\text{V}\left(\theta ,\overset{\cdot }{\theta }\right)$ denotes the vector of centrifugal and Coriolis torque. $\tau_{{\text{f}}_{}}\left(\theta ,\overset{\cdot }{\theta }\right)$ is the friction vector of the robot system. **D**_u_ represents the lumped effects of uncertainties. $\Gamma_{1}^{}$ and **Γ**_**2**_ represent the Cartesian interaction forces acting on the upper arm and the end-effector of the exoskeleton, respectively. Jacobian matrixes, **J**_**1**_(**θ**) and **J**_**2**_(**θ**), provide the mapping from the interaction forces to the torques at robot joints, and they can be derived from the kinematics model of the exoskeleton (43). The vector **τ** is the output driving torques of servo motors.

It should be pointed out that the vector of gravitational torque is eliminated in Equation (1), as the proposed exoskeleton robot is kept in a static balanced state during the operation (42). Note that since the movement range of the passive self-aligning platform is fairly limited and small during rehabilitation training, the platform is assumed to be stationary while developing the system dynamic model. The experimental results obtained in our previous work have verified the rationality of this assumption.

The friction existing in the exoskeleton system can be basically subdivided into three parts: the friction from Bowden-cable transmission systems $\tau_{b}\left(\theta ,\overset{\cdot }{\theta }\right)$, the friction from the reducers of motors $\tau_{r}\left(\theta ,\overset{\cdot }{\theta }\right)$, and the friction from robot joints $\tau_{j}\left(\theta ,\overset{\cdot }{\theta }\right)$. Thus, we have

(2) $$\tau_{\text{f}}(\theta ,\dot{\theta })=\tau_{\text{b}}(\theta ,\dot{\theta })+\tau_{\text{r}}(\theta ,\dot{\theta })+\tau_{\text{j}}(\theta ,\dot{\theta }).$$

The friction of the Bowden-cable transmission can be derived from the torque transmission model presented in our previous research (41). The relation between the motor-driving torque acting on the *i*th proximal pulley, i.e., τ_*in, i*_, and the output torque acting on the *i*th robot joint, i.e., τ_*out, i*_, can be described using the following equation:

(3) $$\tau_{in,i}=\frac{r_{1}}{r_{2}}\tau_{out,i}+\text{sign}({\dot{\theta }}_{i})2T_{i}r_{1}\lambda_{i}\mu_{i}$$

where *T*_*i*_ and λ_*i*_ are the cable pretension and the bending angle of the *i*th Bowden-cable unit. The term *μ*_*i*_ represents the Coulomb friction coefficient. *r*_1_ and *r*_2_ denote the radiuses of the proximal pulley and the distal pulley. Note that the values of *r*_1_ and *r*_2_ of the presented exoskeleton are equal to 25 mm. Thus, Equation (3) can be refined as

(4) $$\tau_{in,i}-\tau_{out,i}=\tau_{b,i}=\text{sign}({\dot{\theta }}_{i})2T_{i}r_{1}\lambda_{i}\mu_{i},$$

where τ_*b*__, i_ is the friction torque from the *i*th Bowden-cable transmission unit.

According to the existing research results (44), the friction of the motor reducers and robot joints can be simplified as a Coulomb-viscous model as follows:

(5) $$\tau_{r,i}+\tau_{j,i}=b_{i}{\dot{\theta }}_{i}+\text{sign}({\dot{\theta }}_{i})\tau_{c,i},$$

where τ_*r*__, i_ and τ_*j*__, i_ represent the friction torques from the *i*th reducer and robot joint. *b*_*i*_ is the coefficient of viscosity. τ_*c, i*_ denotes the Coulomb friction torque.

Therefore, by inserting Equations (4, 5) into Equation (2), the *i*th element of the integrated system friction vector can be obtained and expressed as

(6) $$\tau_{f,i}=b_{i}{\dot{\theta }}_{i}+\text{sign}({\dot{\theta }}_{i})\left[2T_{i}r_{1}\lambda_{i}\mu_{i}+\tau_{c,i}\right]=b_{i}{\dot{\theta }}_{i}+\text{sign}({\dot{\theta }}_{i})\tau_{e,i}.$$

Here, τ_*e*__, i_ represents the equivalent parameter for the Coulomb friction torque.

### Patient-active admittance control

**Figure 4:**
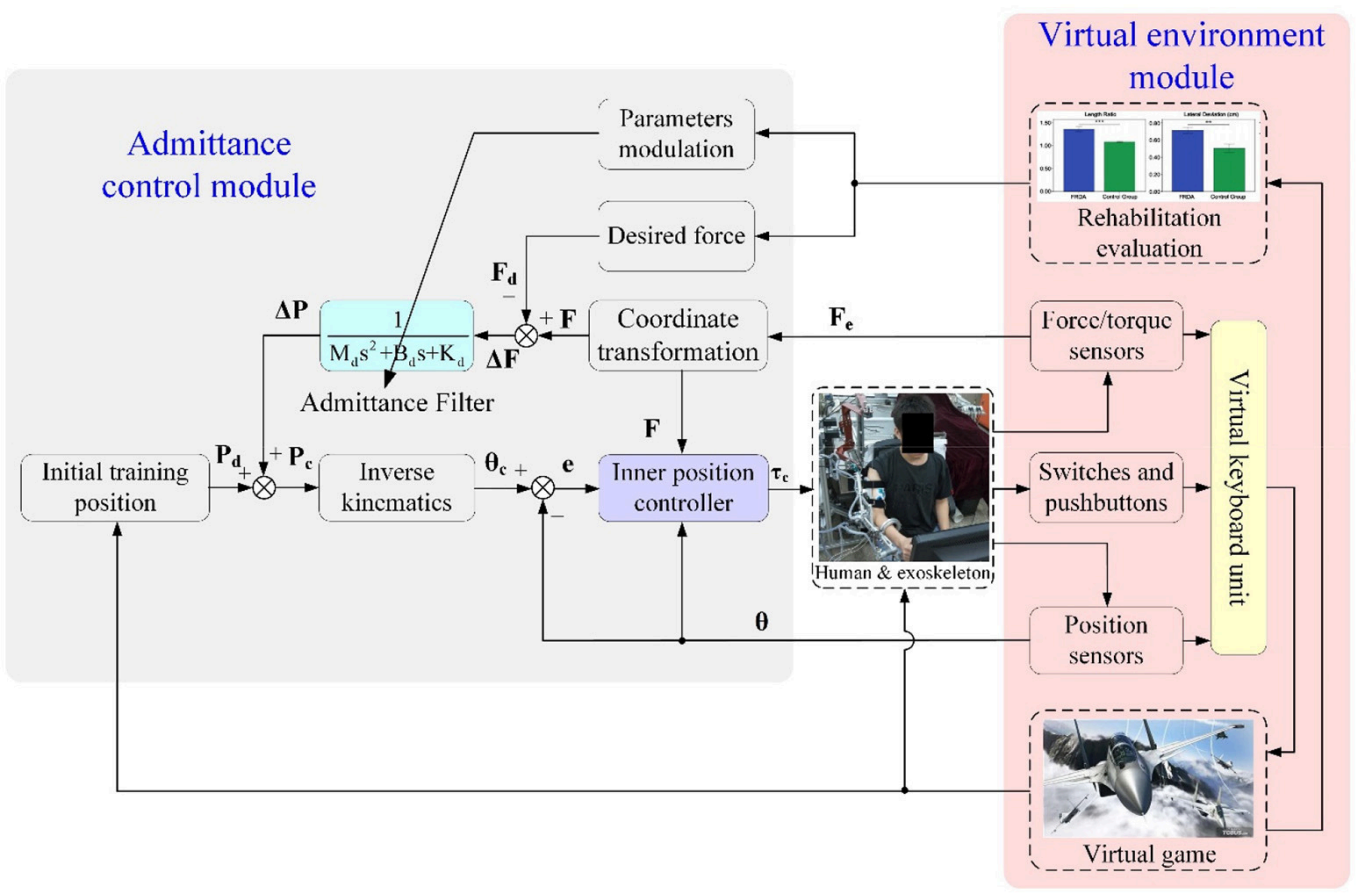
Overall block diagram of the proposed admittance-based patient-active control strategy.

Robot-assisted rehabilitation training with games played in virtual reality environments has positive effects on inducing active patient participation and encouraging the neural plasticity of the brain (36). Thus, in our research, a patient-active training strategy is developed by integrating an admittance control module and a virtual environment module. The overall block diagram describing the major components of the proposed control scheme is depicted in Figure 4. The exoskeleton system is equipped with a graphical guidance interface running on a commercial computer with different virtual games. During rehabilitation training, the operator was seated on a chair with the upper arm connected to the exoskeleton via a custom-made cuff and the palm grasping the end-effector. Instructions were presented on the screen placed in front of the therapeutic robot. The visual and acoustical feedbacks from the virtual environment provide effective guidance for the operator to enhance the awareness of training performance and execute the required training tasks. The system can provide quantitatively evaluated reports for proposed exercises. The statistical training results facilitate therapists to modulate the exercise difficulty levels and training intensity based on the practical recovery status of patients and therapeutic goals.

The real-time information about robot configuration and human–robot interaction, which are acquired from F/T sensors, position sensors, as well as the switches and pushbuttons mounted at the end-effector, are transmitted into a virtual keyboard unit to handle the virtual game process. The virtual keyboard unit was developed in the Microsoft Visual C++ programming environment and used as the interface between the operator and the virtual environment, allowing patient-active training during rehabilitation. In other words, the disabled patient can play the virtual game by actively manipulating the exoskeleton, and this is beneficial to the recovery of motor function and mental confidence. Communication between the RTW control system and the virtual environment was established via the transmission control protocol/internet protocol (TCP/IP).

The admittance control module is developed to regulate the robot configurations and human–robot interaction forces at the end-effector while executing patient-active training. The fed-back forces from the end-effector, **F**_e_(*t*), measured by the distal F/T sensor are transformed into the actual forces in basic Cartesian space, **F**(*t*), through coordinate conversion. The force errors, Δ**F**(*t*), between the desired interaction forces, **F**_d_(*t*), and the actual interaction forces at the end-effector are transmitted into an admittance filter to compute the value of position regulation, Δ**P**(*t*). The desired impedance characteristic describing the relationship between interaction force and end-effector position can be expressed as

(7) $$\Delta F(t)=M_{\text{d}}\Delta \overset{..}{P}(t)+B_{\text{d}}\Delta \dot{P}(t)+K_{\text{d}}\Delta P(t),$$

where

$$\{\begin{array}{c} \Delta F(t)=F(t)-F_{\text{d}}(t)\\ \Delta P(t)=P_{\text{c}}(t)-P_{\text{d}}(t) \end{array}$$

Here **P**_d_ (*t*) denotes the desired inertial training position of the end-effector predefined according to the virtual game scenes; **P**_c_ (*t*) denotes the corresponding control position of the end-effector in Cartesian space; **M**_d_, **B**_d_, and **K**_d_ are the objective inertia, damping, and stiffness of the admittance filter.

Thus, in the frequency domain, Equation (7) can be represented as

(8) $$\frac{\Delta F(s)}{M_{\text{d}}s^{2}+B_{\text{d}}s+K_{\text{d}}}=\Delta P(s).$$

The resultant control position, **P**_c_ (*t*), which depends on the distal interaction forces and admittance parameters, can be converted to the angular position in joint coordinates, θ_c_ (*t*), via the inverse kinematics of the robot. A detailed description of the analytical inverse kinematic resolution of the proposed redundant exoskeleton has been analyzed in (43).

Thus, from Equations (7, 8), we have

(9) $$\text{Inv}\left(P_{\text{d}}(t)+\Delta P(t)\right)=\text{Inv}\left(P_{\text{c}}(t)\right)=\theta_{\text{c}}(t),$$

where Inv (·) denotes the inverse kinematic calculation. The inner position controller is implemented to move the robot to the desired position expressed by θ_c_ (*t*). The position control was realized by using a fuzzy sliding mode control (FSMC) scheme developed based on the system dynamics model, and it was proposed in our previous research (45).

According to Equation (1), since the inertia matrix is symmetric and positive definite, the dynamic model is refined as

(10) $$\overset{..}{\theta }=M{\left(\theta \right)}^{-1}\left[\tau -V(\theta ,\dot{\theta })-\tau_{\text{f}}(\theta ,\dot{\theta })-D_{\text{u}}+J_{1}^{T}(\theta )\Gamma_{1}+J_{2}^{T}(\theta )\Gamma_{2}\right].$$

The switching function of sliding surface **S**(*t*) can be defined as

(11) $$S(t)=\lambda e(t)+\eta \dot{e}(t),$$

where **e**(*t*) = **θ**_c_(*t*) − **θ**(*t*) denotes the position tracking error. $\overset{\cdot }{\text{e}}\left(t\right)={\overset{\cdot }{\theta }}_{\text{c}}\left(t\right)-\overset{\cdot }{\theta }\left(t\right)$ denotes the velocity tracking error. **λ** and **η** represent the positive diagonal matrixes of proportional gain and derivative gain.

Combining Equations (10, 11), the deviation of the sliding variable can be calculated as

(12) $$\begin{array}{l} \dot{S}(t)=\lambda \dot{e}(t)+\eta {\overset{..}{\theta }}_{\text{c}}(t)-\eta \text{M}{\left(\theta \right)}^{-1}[\tau -V(\theta ,\dot{\theta })-\tau_{\text{f}}(\theta ,\dot{\theta })-D_{\text{u}}\\ \;+J_{1}^{T}(\theta )\Gamma_{1}+J_{2}^{T}(\theta )\Gamma_{2}], \end{array}$$

And then, the control law can be designed as

(13) $$\begin{array}{l} u(t)=M(\theta )\eta^{-1}\lambda \dot{e}(t)+M(\theta ){\overset{..}{\theta }}_{c}(t)+V(\theta ,\dot{\theta })+\tau_{\text{f}}(\theta ,\dot{\theta })\\ \;-J_{1}^{T}(\theta )\Gamma_{1}-J_{2}^{T}(\theta )\Gamma_{2}+\varepsilon M(\theta )\eta^{-1}\text{sgn}(S), \end{array}$$

where sgn (·) means the sign function. ε represents a positive switching gain computed by a fuzzy controller (45).

For the purpose of demonstrating the stability of the proposed patient-active control algorithm, a positive definite Lyapunov function candidate is defined as

(14) $$V=\frac{1}{2}S^{\text{T}}(t)S(t).$$

Differentiating Equation (14) with respect to time *t* and combining Equations (10) with Equation (13) yields Equation (15) as follows.

(15) $$\begin{array}{l} \dot{V}=S^{\text{T}}(t)\dot{S}(t)\\ \;=S^{\text{T}}(t)[\lambda \dot{e}(t)+\eta {\overset{..}{\theta }}_{\text{c}}(t)-\eta M{\left(\theta \right)}^{-1}[u(t)-V(\theta ,\dot{\theta })\\ \;-\tau_{\text{f}}(\theta ,\dot{\theta })-D_{\text{u}}+J_{1}^{T}(\theta )\Gamma_{1}+J_{2}^{T}(\theta )\Gamma_{2}]]\\ \;=S^{\text{T}}(t)[\lambda \dot{e}(t)+\eta {\overset{..}{\theta }}_{\text{c}}(t)-\eta M{\left(\theta \right)}^{-1}[M(\theta )\eta^{-1}\lambda \dot{e}(t)\\ \;+M(\theta ){\overset{..}{\theta }}_{\text{c}}(t)-D_{\text{u}}+\varepsilon M(\theta )\eta^{-1}\text{sgn}(S)]]\\ \;=S^{\text{T}}(t)[-\eta M{\left(\theta \right)}^{-1}(\varepsilon M(\theta )\eta^{-1}\text{sgn}(S)-D_{\text{u}})]\\ \;=S^{\text{T}}(t)\eta M{\left(\theta \right)}^{-1}D_{\text{u}}-\varepsilon S^{\text{T}}(t)\text{sgn}(S)\\ \;=S^{\text{T}}(t)\eta M{\left(\theta \right)}^{-1}D_{\text{u}}-\varepsilon \sum_{i\;=\;1}^{6}\left|S_{i}\right| \end{array}$$

Here, the lumped effects of uncertainties **D**_u_ are supposed to satisfy the following assumption.

*Assumption 1:* The boundary of the uncertainty term can be given as (46)

(16) $$\left|D_{u}\right|<\gamma .$$

If the positive switching gain is selected as

(17) $$\varepsilon >\left|\eta \text{M}{\left(\theta \right)}^{-1}D_{\text{u}}\right|,$$

then (16) can be rewritten as

(18) $$\dot{V}=S^{\text{T}}(t)\eta \text{M}{\left(\theta \right)}^{-1}D_{\text{u}}-\varepsilon \sum_{i\;=\;1}^{6}\left|S_{i}\right|<\varepsilon \left|S^{\text{T}}(t)\right|-\varepsilon \sum_{i\;=\;1}^{6}\left|S_{i}\right|<0$$

Here, it can be observed that $\overset{\cdot }{V}\left(t\right)$ is negative definite. Thus, the proposed controller satisfies the Lyapunov stability criteria, and the position tracking error gradually converges to the sliding surface *S*(*t*) = 0 in finite time.

## Results

Upon completing the hardware design, dynamic modeling, and controller development, three typical experiments were conducted on the exoskeleton to validate the feasibility of the proposed patient-active admittance control strategy. In the first case, the position tracking experiment without the operator and external interactions was carried out to analyze the control performance of the inner fuzzy sliding mode position controller. In the second case, the free arm movement training experiment with different admittance parameters was conducted with three healthy subjects. In the third case, the virtual airplane-game operation training with graphical guidance was carried out with two healthy subjects and eight stroke patients with different Fugl-Meyer assessment (FMA) scores (47). The ethical approval of the implemented experimental approaches have been obtained from the Human Subjects Ethics Subcommittee of the Nanjing University of Aeronautics and Astronautics. The participants of this research were recruited from the local districts through advertisement. The research on the developed patient-active control strategy had been conducted since February 2017. The upper limb rehabilitation exoskeleton system was designed by Qingcong Wu and Xingsong Wang.

### Position tracking experiments

According to the control diagram illustrated in Figure 4, the proposed admittance-based patient-active control scheme will be converted into a position controller to track the predefined training trajectory, if the desired force and the actual human–robot interaction force are equal to zero. Actually, the inner position controller is one of the kernel algorithms of the patient-active control method, as it is responsible for modulating the exoskeleton configuration based on the position regulation value output from the admittance filter. Therefore, it is necessary to demonstrate the feasibility of the proposed FSMC-based inner position control strategy.

**Figure 5:**
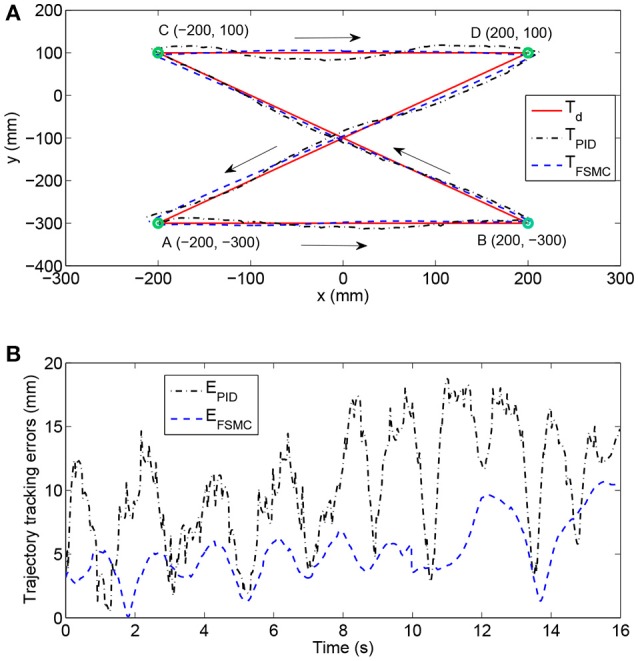
Experimental results of position tracking tests with different control schemes. **(A)** The comparison among desired trajectory, the trajectory with PID controller, and the trajectory with fuzzy sliding mode controller (FSMC). **(B)** Time histories of position tracking errors.

The position tracking experiment without the participation of the subject was carried out to evaluate the position control performance. The desired position trajectory of the end-effector, which is defined in the base robot coordinate frame of the exoskeleton and parallel to the coronal plane, is shown in Figure 5A. There are four waypoints (A, B, C, and D) defined in the available workspace of the exoskeleton, and the initial location of the end-effector is set to Point A. The overall trajectory can be divided into four continuous subtrajectories. Firstly, the end-effector is controlled to move horizontally from point A to point B in 4 s. It also takes 4 s to move from point B to point C. The third subtrajectory is designed to follow the path from point C to point D in 4 s. Finally, the end-effector is returned from point D to point A in the last 4 s. It should be pointed out that, to guarantee the smoothness of the trajectory and avoid undesirable impact phenomena, the subtrajectory between two points is determined based on the minimum jerk cost principle (48). The terminal motion states of the former subtrajectory, including the positions, velocities, and accelerations, are the same as the initial motion states of the next subtrajectory.

The experimental performance of the proposed FSMC algorithm was compared to that of a conventional PID controller. The selected control parameters of the PID controller (i.e., proportional gain, integral gain, and derivative gain) were estimated via the Ziegler–Nichols method and carefully optimized by trial and error. The control parameters of the FSMC algorithm were optimally adjusted by intensive tests to improve control accuracy and ensure system stability. The results of trajectory tracking experiments with different control strategies are compared in Figure 5A. In addition, the time histories of position tracking errors, which are defined as the absolute distance between the desired and actual coordinates of the end-effector, are illustrated in Figure 5B. As can be observed, the tracking performance of the FSMC scheme has turned out to be far more effective than that of the PID controller. More specifically, the average output-tracking error declines from 10.42 mm (PID) to 5.21 mm (FSMC), while the root-mean-square error (RMSE) declines from 11.56 mm (PID) to 5.78 mm (FSMC) during the experiments. The peak-to-peak error is smaller (10.15 mm) with the FSMC strategy in comparison with that with the PID algorithm (18.75 mm). Therefore, the obtained results of position tracking experiments confirm the validity of the inner position controller.

### Free arm training experiment

The fundamental purpose of developing the patient-active control framework is to encourage the voluntary efforts and active participations of patients during rehabilitation training. The basic function of the admittance control module, as the core component of the patient-active controller, is to modulate the configuration of the exoskeleton according to the detected real-time human–robot interaction force and the desired mechanical admittance characteristic. Hence, the second experiment was conducted to evaluate the feasibility of the admittance controller in free arm operation training.

In this experiment, three healthy neurologically intact volunteers with various anthropometric parameters and ages (subject H1: male, height/1.7 m, weight/62.5 kg, age/29 years; subject H2: male, height/1.76 m, weight/66.2 kg, age/22 years; subject H3: female, height/1.62 m, weight/51.8 kg, age/46 years) were asked to undergo free arm movement trainings with different admittance parameters. The volunteers were comfortably positioned in the therapeutic system with their right arms wearing the exoskeleton. The limb lengths and the joint axes of the upper arm and the forearm of the exoskeleton were adjusted in accordance with the anthropometric parameters of wearers. Next, the subjects grasped the end-effector of the exoskeleton and actively performed horizontal reciprocating movements along the x-axis of the base coordinate system. The relation between the interaction force, measured by the distal F/T sensor of the end-effector, and the position deviation Δ**x**, calculated from the positions of the robot's joints and inverse kinematics, reflects the corresponding training difficulty and intensity. The range of position deviation was generally restricted within the interval of [−400, 400 mm].

To compare and investigate the training performance with different admittance parameters, three different groups of parameters (E1, E2, and E3) were implemented to the admittance filter during the free arm movement experiments, as shown in Table 1. The subjects were required to repeat the aforementioned training task six times for each condition.

**Table 1:** Admittance parameters of different working conditions.

**Conditions**	**M_d_ (Ns^2^/mm)**	**B_d_ (Ns/mm)**	**K_d_ (N/mm)**
E1	diag [0.060, 0.060, 0.060]	diag [0.060, 0.060, 0.060]	diag [0.060, 0.060, 0.060]
E2	diag [0.025, 0.025, 0.025]	diag [0.025, 0.025, 0.025]	diag [0.025, 0.025, 0.025]
E3	diag [0.015, 0.015, 0.015]	diag [0.015, 0.015, 0.015]	diag [0.015, 0.015, 0.015]

Existing study results indicate that the EMG signals provide information on the scale of muscular power and activation patterns (49). Besides, the results from our previous tests show that the EMG signal strength from the bicipital muscle and the brachioradialis muscle were larger than those from the brachialis muscle and the triceps muscle during free arm operation. Therefore, to analyze and compare the training intensity with different admittance parameters, the surface EMG signals from the bicipital and brachioradialis muscles of the wearers were acquired during the free arm training process. The measurement system mainly consists of active bipolar surface electrodes and sensors (Sunlephant-6 EMG System, Sichiray) used to obtain original signals, an integrated processing unit (SHIELD-EKG, Olimex) used for signal acquisition, amplification, and filtering, and a data analysis system running the Microsoft Visual Studio environment used for offline analysis. Before data acquisition, the exposed skin areas over the bicipital muscle and the brachioradialis muscle were cleaned with an alcohol pad to decrease contact resistance. The surface electrodes, with an effective area of 10 × 10 mm for each unit, were attached to the skin via double-faced adhesive tape. The EMG signals were sampled at 1 kHz with 12-bit resolution and filtered by using a third-order Butterworth high-pass filter with a cut-off frequency of 20 Hz. The processed signals were wirelessly transmitted to the data analysis system via a Bluetooth network.

**Figure 6:**
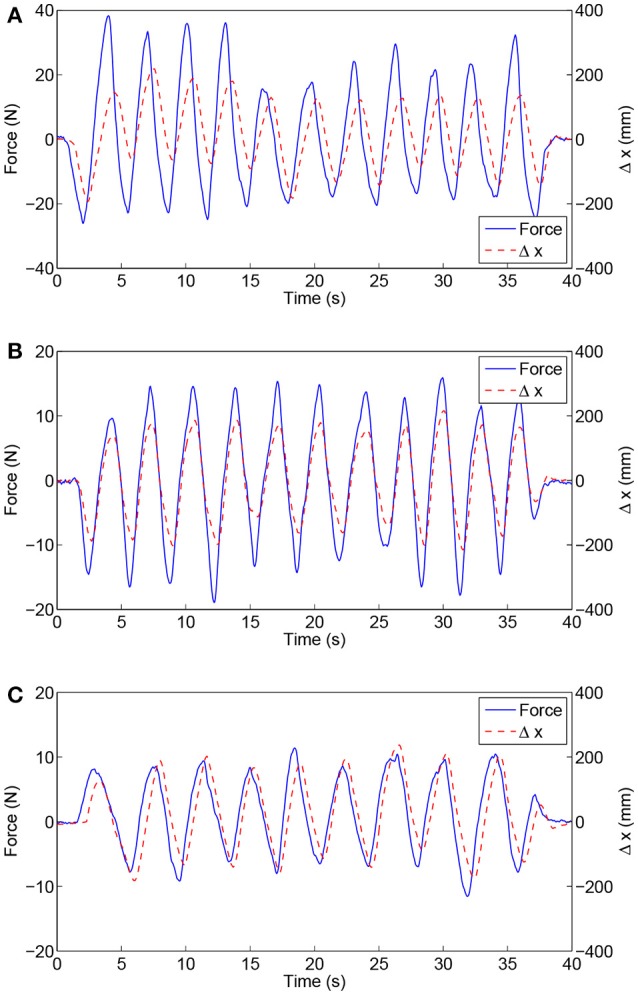
Experimental results of free arm training tests conducted with healthy subject H1 with different admittance parameters. **(A)** Results under condition E1. **(B)** Results under condition E2. **(C)** Results under condition E3.

**Figure 7:**
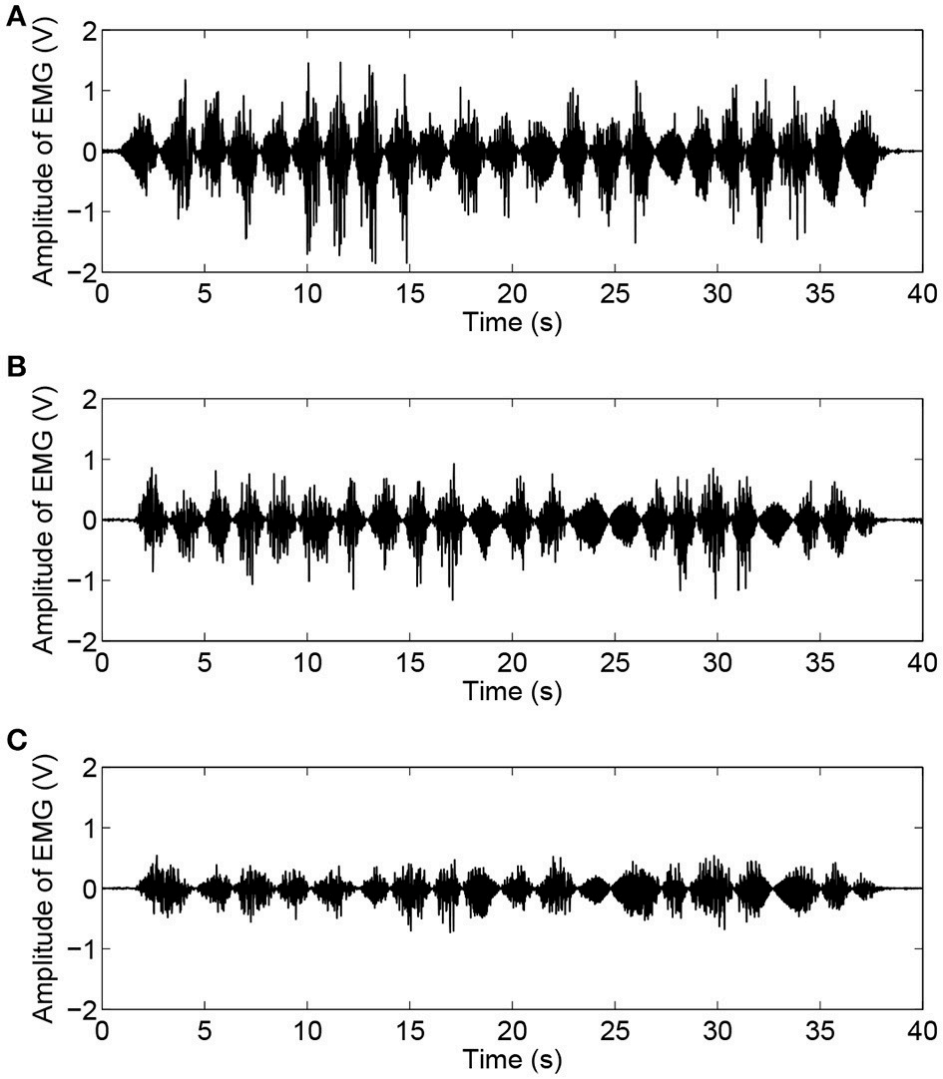
Time histories of EMG activity levels of bicipital muscle during free arm training tests conducted with healthy subject H1 for different admittance parameters. **(A)** EMG signals under condition E1. **(B)** EMG signals under condition E2. **(C)** EMG signals under condition E3.

As shown in Figure 6, the experimental results of subject H1 (single trial) present the relationships between the interaction force and position variation with different admittance parameters. It can be clearly observed that during the patient-active operation experiments, the changing tendency of position deviation is basically consistent with that of the interaction force. The active forces, from the subject and applied to the end-effector, lead to a corresponding modulation of robot configuration and, as a result, realize the active participation in rehabilitation training. Furthermore, increases in admittance parameters cause larger interaction forces for the same free arm training task. Figure 7 compares the EMG activity levels of subject H1 under different experimental conditions. The RMS approach is used for the feature extraction of raw EMG signals in this research. The RMS EMG value of the bicipital muscle under experimental condition E1 (0.466 V) turned out to be larger than those under condition E2 (0.281 V) and condition E3 (0.165 V).

**Figure 8:**
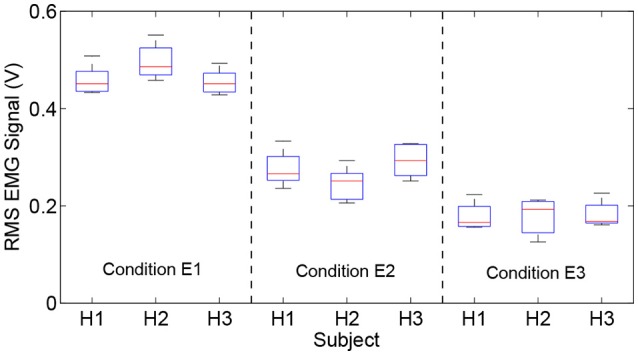
Comparison results of RMS EMG values of bicipital muscles for different subjects and admittance parameters.

Moreover, the experimental results (six trials) of the RMS EMG values from the bicipital muscle of different subjects and admittance parameters are compared in Figure 8. It can be concluded that the increase in the admittance parameters of the proposed patient-active control scheme leads to the increase of EMG activity levels. However, the specific surface EMG activity levels vary from person to person during the same experiment. The statistical results for the bicipital muscle and brachioradialis muscle are compared in Table 2. The experimental results indicate that by modulating the admittance filter, motion resistance and training intensity during patient-active rehabilitation therapy can be rationally adjusted to meet the capabilities and requirements of different patients.

**Table 2:** Comparison of statistical results of RMS EMG values of bicipital muscle and brachioradialis muscle under different experimental conditions.

**Con**	**RMS EMG values of different subjects (V)**											
	**H1**				**H2**				**H3**			
	**Biceps**		**Brachioradialis**		**Biceps**		**Brachioradialis**		**Biceps**		**Brachioradialis**	
	**Max**	**Med**	**Max**	**Med**	**Max**	**Med**	**Max**	**Med**	**Max**	**Med**	**Max**	**Med**
E1	0.51	0.45	0.46	0.40	0.55	0.48	0.50	0.43	0.49	0.46	0.42	0.37
E2	0.33	0.27	0.30	0.24	0.29	0.25	0.35	0.25	0.32	0.30	0.28	0.21
E3	0.22	0.16	0.21	0.15	0.21	0.19	0.26	0.20	0.22	0.17	0.19	0.15

*Con, condition; Max, maximum value; Med, median value*.

### Virtual airplane-game operation experiment

**Figure 9:**
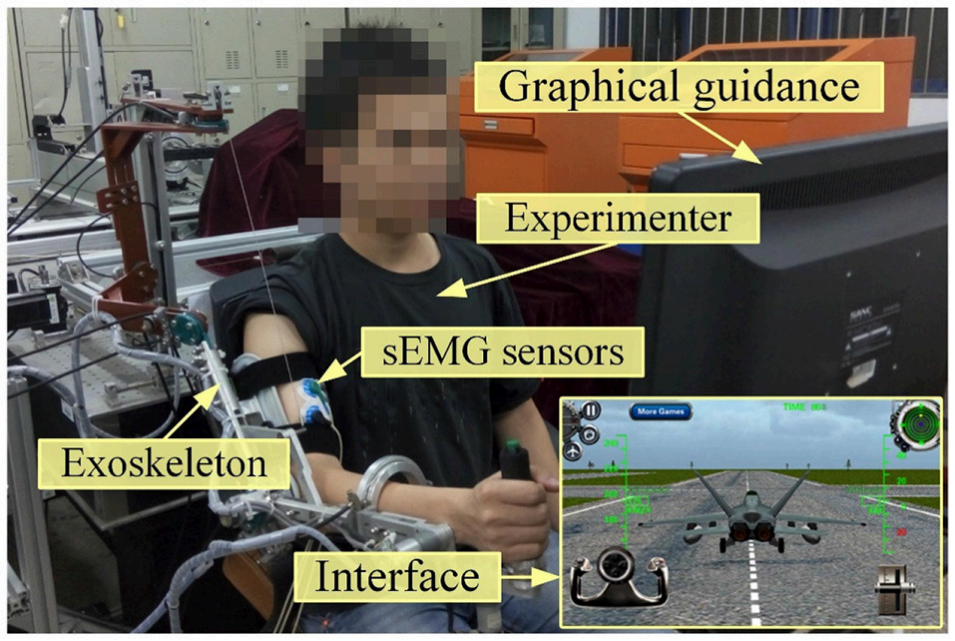
Experimental scenarios of a healthy volunteer conducting virtual airplane-game operation experiment.

The purpose of the third experiment was to evaluate the practical feasibility by applying the proposed admittance-based patient-active controller to rehabilitation training in a virtual reality environment. Figure 9 presents the experimental scenarios in which a healthy volunteer wearing an exoskeleton actively plays the virtual airplane-game with visual and acoustical feedback instructions. There were ten volunteers (two healthy subjects and eight post-stroke chronic patients) with various FMA scores participating in the virtual airplane-game operation experiment. The detailed anthropometry parameters and the motor ability of each subject are presented in Table 3.

**Table 3:** Characteristics of the subjects participating in experiments.

**Sub**	**Gender**	**Age (years)**	**Height (m)**	**Weight (kg)**	**FMA score (upper limb)**	**FMA score (shoulder)**	**FMA score (wrist)**	**FMA score (hand)**	**Status**
H1	Male	26–30	1.70	62.5	66 (full)	16	10	22	Healthy
H2	Male	21–25	1.76	66.2	66 (full)	16	10	22	Healthy
P1	Male	61–65	1.73	66.2	60	15	8	21	Slight paralysis
P2	Female	36–40	1.56	47.2	58	14	8	20	Slight paralysis
P3	Female	46–50	1.71	62.6	51	12	7	18	Moderate paralysis
P4	Male	56–60	1.70	66.5	49	13	6	18	Moderate paralysis
P5	Male	66–70	1.82	75.3	41	11	5	16	Moderate paralysis
P6	Male	41–45	1.76	62.0	38	10	6	15	Moderate paralysis
P7	Male	46–50	1.79	64.7	16	5	3	4	Severe paralysis
P8	Female	51–55	1.70	61.5	8	2	2	2	Severe paralysis

During the trial, the participants were required to actively manipulate the exoskeleton system and control the virtual airplane prototype displayed on the screen to accomplish the requested actions along the predesigned trajectory, including the taking-off operation, landing operation, and free-flight operation. To modulate the sensitivity of game control and improve the maneuverability of virtual training, the predefined thresholds of position variation (100 mm) and interaction force (10 N) were integrated into the virtual keyboard program. Only if the position variation and detected force exceed the respective threshold simultaneously, the virtual keyboard unit will trigger the corresponding keys to regulate the game process. In this way, the virtual keyboard acts as the interface that maps the human–robot interaction force and the robot configuration to the operations in the airplane game.

**Figure 10:**
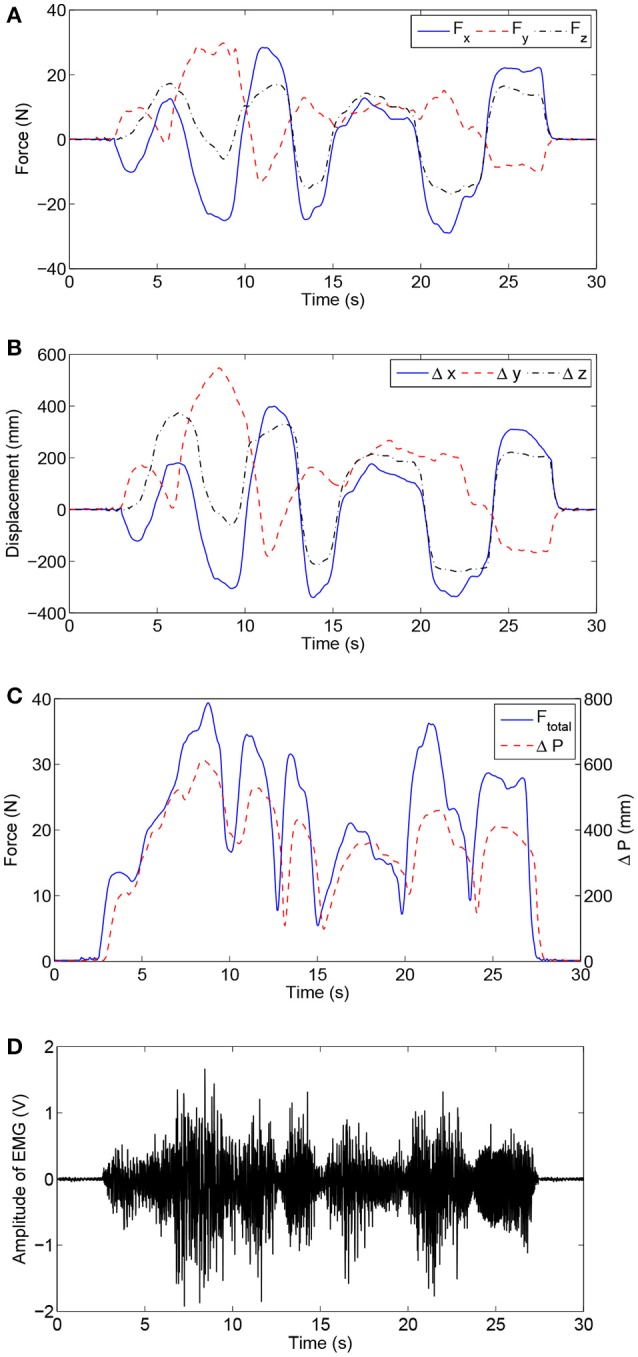
Experimental results of virtual airplane-game operation training executed by healthy subject H1. **(A)** Interaction forces in different directions. **(B)** Position deviations in different directions. **(C)** Comparisons of the resultant interaction force and combined position deviation. **(D)** Time history of EMG activity levels of bicipital muscle.

In this experiment, the admittance parameters were set as follows: **M**_d_ = diag [0.03, 0.04, 0.06] Ns^2^/mm, **B**_d_ = diag [0.05, 0.04, 0.08] Ns/mm, **K**_d_ = diag [0.05, 0.06, 0.08] N/mm. All the subjects were required to make their best efforts to complete the airplane operation task six times. Figure 10 presents the experimental results of virtual airplane-game operations performed by healthy subject H1 in a single trial. More specifically, Figures 10A,B present the comparisons of interaction forces and position deviations in different directions of the base coordinate system. Further, the resultant interaction forces and combined position deviations are compared in Figure 10C. It can be seen that the variation tendencies of human–robot interaction forces are consistent with those of position deviations. The time history of the amplitude of EMG signals is depicted in Figure 10D, and the calculated RMS EMG value of the bicipital muscle is ~0.407 V. The subject wearing the exoskeleton system was able to actively handle the motion of the virtual airplane during rehabilitation training. Thus, experimental results substantiate the feasibility of the proposed control strategy.

**Figure 11:**
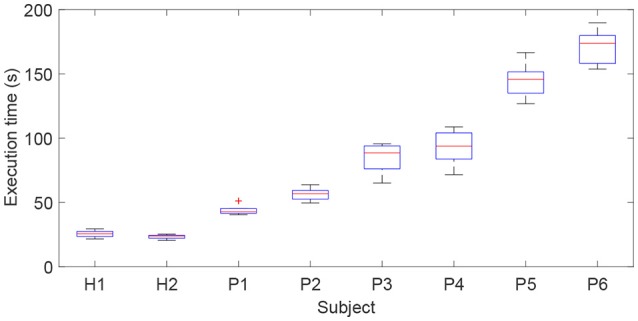
Comparison results of execution times for subjects with different FMA scores required to complete the virtual airplane-game operation task.

The execution time required to finish the airplane operation task reflects the difficulty of the game for a specific participant. The execution times for the subjects with different FMA scores were recorded and compared (Figure 11). The average execution time for each subject was obtained (H1: 25.6 s; H2: 23.9 s, P1: 42.7 s; P2: 56.8 s; P3: 88.6 s; P4: 93.2 s; P5: 145.3 s; P6: 173.5 s). It should be pointed out that subjects P7 and P8 with severe paralysis were not able to complete the required training task and hence their data were excluded from the statistical data. The experimental results show that, with the proposed exoskeleton system and patient-active control strategy, a subject with adequate motor function capacity is able to actively handle the motion of virtual airplane and complete the requested training task in the virtual environment. The required execution time increases with decreasing motor function capacity of patients. The active participation of patients can be encouraged. However, the developed patient-active control scheme is not suitable for severely paralyzed patients, who require passive repetition training with the assistance of an exoskeleton.

## Discussion

From the results of three different experiments—the position tracking experiment, the free arm training experiment, and the virtual airplane-game operation experiment—it can be observed that the proposed FSMC-based admittance control strategy is capable of providing patient-active mode training during rehabilitation. More specifically, from the results of position tracking tests, it can be seen that the inner FSMC position controller is capable of achieving smaller tracking errors and better control performance when compared with the conventional PID controller, and can ensure the modulation accuracy of robot configuration based on the output value of the admittance filter. From the results of the free arm training experiment conducted with three healthy participants with different admittance parameters, it can be observed that the developed FSMC-based admittance controller can assist the patient actively undergo the free arm movement trainings with desired training difficulty and intensity. Moreover, it is able to encourage the voluntary efforts and active participations of patients during rehabilitation training. The increase of the admittance parameters of patient-active controller leads to the increase of motion resistance and the EMG activity levels of the bicipital muscle. According to the results of virtual airplane-game operation experiment conducted with two healthy volunteers and eight post-stroke chronic patients with various FMA scores, it can be found that, with the developed admittance-based patient-active control strategy, the volunteer wearing the exoskeleton system is able to actively handle the motion of virtual airplane during rehabilitation training. The rehabilitation training integrated with commercial virtual games without a specific predefined trajectory has valid impact on enhancing the interestingness of physical therapy and, furthermore, contributes to neurological rehabilitation and psychotherapy. The experimental results for different disabled patients indicate that the required execution time of the same operation task increases with the decrease of motor functions of patients. Besides, the feasibility in encouraging active participation has been proved from the statements of volunteers. In the actual performance, the protocol for each patient should be selected based on several factors, such as the detailed FMA scores of patient, the desired rehabilitation training programs, the quantitatively statistical training reports from the evaluation system, and the experience of physiotherapists.

### Limitations

There are several limitations in the proposed exoskeleton and control strategy. Firstly, the proposed patient-active controller can be used with patients with good cognitive function, fair sitting balance, and good upper limb motor performance; it cannot be applied to severely paralyzed subjects without any motor functions. The resistance from the exoskeleton needs to be overcome during patient-active training. Secondly, the proposed exoskeleton can be used only for patients with right-sided weakness, thus limiting the range of applicability. An extension of this research will be to improve the control strategy to be suitable for severely paralyzed patients. The mechanical structure will be further optimized to realize the switch between right arm training and left arm training. The effectiveness of the proposed rehabilitation exoskeleton system and control strategy will be tested and demonstrated via a more substantial trial in future works. Besides, the perceived value and motivation of patient will be qualitatively evaluated by using electroencephalography (EEG) sensor. A rational rehabilitation evaluation system will be developed to estimate recovery progress. The controller parameters, such as the admittance filter, should be optimized based on the evaluation of patients to improve treatment efficiency. In addition, the therapeutic training of the human hand will be integrated into the rehabilitation system.

## Conclusion

This paper dealt with the development and validation of an upper limb exoskeleton and a patient-active admittance control strategy, which can be applied to assist the patients with affected arm motor functions in performing active training in virtual environments. Firstly, the major hardware of the wearable exoskeleton system, including the gravity-balanced mechanical structure with 7 + 2 DOFs and the MATLAB/RTW real-time control system, was briefly introduced. Then, the system dynamic model was established using the Lagrangian method. To encourage the active participation of patients during rehabilitation training, a patient-active controller was proposed based on the dynamic model. A patient wearing the exoskeleton can undergo active rehabilitation training with the guidance of a graphical interface, and the training trajectory is not constrained to a specific predetermined trajectory. Different kinds of existing commercial virtual games, which are far more realistic and interesting than the simple self-designed ones, can be integrated into the proposed control strategy via a virtual keyboard unit. The feasibilities of the proposed therapeutic system and control strategy were verified via three pilot tests. A position tracking experiment was carried out to evaluate the performance of the inner fuzzy sliding mode position control strategy. A free arm training experiment was conducted with three healthy subjects to study the functionality of the admittance module during free arm operation. To verify the practical feasibility of applying the patient-active control strategy to the training within virtual environments, a virtual airplane-game operation experiment was conducted with ten volunteers with different FMA scores.

## Author contributions

QW and XW participated in the design of this study. QW and BC carried out the experiments and analyzed the experimental data. HW contributed in the exoskeleton development and maintenance. QW wrote and modified the manuscript. All authors read and approved the manuscript.

### Conflict of interest statement

The authors declare that the research was conducted in the absence of any commercial or financial relationships that could be construed as a potential conflict of interest.
